# Word Segmentation Cues in German Child-Directed Speech: A Corpus Analysis

**DOI:** 10.1177/0023830920979016

**Published:** 2021-01-30

**Authors:** Katja Stärk, Evan Kidd, Rebecca L. A. Frost

**Affiliations:** Language Development Department, Max Planck Institute for Psycholinguistics, The Netherlands; Language Development Department, Max Planck Institute for Psycholinguistics, The Netherlands; Research School of Psychology, The Australian National University, Australia; ARC Centre of Excellence for the Dynamics of Language, Australia; Language Development Department, Max Planck Institute for Psycholinguistics, The Netherlands

**Keywords:** Language acquisition, speech segmentation, distributional cues, child-directed speech, German

## Abstract

To acquire language, infants must learn to segment words from running speech. A significant body of experimental research shows that infants use multiple cues to do so; however, little research has comprehensively examined the distribution of such cues in naturalistic speech. We conducted a comprehensive corpus analysis of German child-directed speech (CDS) using data from the Child Language Data Exchange System (CHILDES) database, investigating the availability of word stress, transitional probabilities (TPs), and lexical and sublexical frequencies as potential cues for word segmentation. Seven hours of data (~15,000 words) were coded, representing around an average day of speech to infants. The analysis revealed that for 97% of words, primary stress was carried by the initial syllable, implicating stress as a reliable cue to word onset in German CDS. Word identity was also marked by TPs between syllables, which were higher within than between words, and higher for backwards than forwards transitions. Words followed a Zipfian-like frequency distribution, and over two-thirds of words (78%) were monosyllabic. Of the 50 most frequent words, 82% were function words, which accounted for 47% of word tokens in the entire corpus. Finally, 15% of all utterances comprised single words. These results give rich novel insights into the availability of segmentation cues in German CDS, and support the possibility that infants draw on multiple converging cues to segment their input. The data, which we make openly available to the research community, will help guide future experimental investigations on this topic.

## 1 Introduction

One of the first puzzles that children must solve during language acquisition is finding boundaries between individual words in speech. However, this is no easy feat, since there are no perfectly reliable cues that learners can draw upon (Aslin et al., 1996; Lehiste, 1970). Instead, children must look to a broad range of imperfect, probabilistic cues (e.g., stress patterns, phonotactic and allophonic regularities, and information about syllable co-occurrences), and use these in combination (Monaghan, 2017). Importantly, each language differs in the availability and likely combination of cues for segmentation, meaning each solution will necessarily be language-specific (see Cutler, 2012). Studying the distribution of cues to segmentation in a variety of different languages is therefore critical for shaping our understanding of whether and how they aid infants’ language acquisition.

There is a substantial literature documenting the prevalence of various particular segmentation cues across different languages, most prominently in European languages such as English (see e.g., Aslin et al., 1996; Brent & Siskind, 2001; Cutler & Carter, 1987; Piantadosi, 2014) and French, (e.g., Shi & Lepage, 2008; see Aslin et al., 1996, and Kabak et al., 2010, for related results from Turkish and French, and see Saksida et al., 2017, for a larger cross-linguistic comparison), though comparatively less is known about the way such cues occur in German. Moreover, there is a notable absence of comprehensive corpus studies seeking to quantify the availability of individual cues in combination in the input. In the current paper, we present one such study of German child-directed speech (CDS). Building upon past research that has focused on single prominent cues to segmentation (e.g., *word stress*: Cutler & Carter, 1987; *transitional probabilities (TPs)*: Saksida et al., 2017; and *single-word utterances*: Brent & Siskind, 2001), we provide a rare comprehensive assessment of a broad range of cues that have been shown to help learners to locate word boundaries in speech, giving a rich overview of the way these cues exist in German CDS. We address each cue that we study in turn below.

### 1.1 Word stress

One well-established cue to word segmentation is stress; the emphasis of a particular syllable within a word over the others. Regular stress patterns in a given language can help mark particular positions within words, and thus can provide a strong indication of word boundaries. For instance, in English, words are typically stressed on the first syllable (Cutler, 1996; Cutler & Norris, 1988), whereas in Hebrew stress usually occurs in a word-final position (Glinert, 1989)—flagging word onset and offset, respectively. Infants’ use of stress as a cue for speech segmentation has been shown to be guided by the basic rhythm of the language being acquired; infants acquiring syllable-timed languages such as French, Italian, and Cantonese (i.e., languages in which each syllable has the same duration) start with segmentation based on the syllable, while infants acquiring stress-timed languages such as English and German (i.e., languages in which stressed syllables are longer and more emphasized than unstressed syllables) break into the speech stream by assuming a trochaic foot (see e.g., Goyet et al., 2010; Nazzi et al., 2006).

Cutler and Norris (1988) proposed that the occurrence of a strong syllable triggers word segmentation in English, with English speakers interpreting this as the onset of a new word (Curtin et al., 2005; Echols et al., 1997; Houston et al., 2004; Jusczyk et al., 1999; Norris et al., 1995). In English, this strategy promises a high success rate, as 90% of content words begin with a strong syllable (Cutler & Carter, 1987). Jusczyk et al. (1999) reported developmental evidence in support of this claim: in a series of experiments, they showed that 7.5 month-old English infants treated strong syllables as indicators for word onset (e.g., interpreting “guiTAR is” as “gui TARis”), and only learnt to segment words following an atypical stress pattern at a later point in development (10.5 months).

Studies in a range of languages have documented infants’ sensitivity to prosodic cues from a very young age (Bull et al., 1984, 1985; Eilers et al., 1984; Spring & Dale, 1977)—perhaps even from birth (Nazzi et al., 1998; Sansavini et al., 1997), with infants developing a preference for the stress pattern of their native language over the course of development (Jusczyk et al., 1993). For German, there is evidence that young infants (around 5 months old) can discriminate between trochaic and iambic stress, showing a preference for trochaic stress over the less common iambic stress pattern (Friederici et al., 2007; Höhle et al., 2009; Tippmann et al., 2015; Weber et al., 2004). Critically, research has shown that infants can use this information to guide word segmentation (Höhle et al., 2001). Here, we examine the precise way in which lexical stress cues are distributed across words in German CDS, providing key evidence for the widely assumed dominant trochaic stress pattern in German.

### 1.2 Transitional Probabilities

Another likely cue to word segmentation is the TP between syllables (Saffran et al., 1996; Saksida et al., 2017). TPs express the likelihood that particular syllables will occur alongside each other in speech, given their prior co-occurrence in the input (both together, and with other items). Languages typically have higher TPs within than between words, such that word boundaries can be inferred at the point at which the subsequent syllable is hard to predict, given the prior syllable. For instance, in the sequence “*pretty baby*” the within-word syllable transitions from “*pre*” to “*ty*” and from “*ba*” to “*by*” have higher TPs (and are therefore easier to predict) than the between-word transition from “ty” to “ba” (Saffran, 2003, reports a TP of 0.8 for the transition from pre to ty compared to a TP of 0.0003 from ty to ba).

In an extensive body of research, learners of all ages have been found to be highly sensitive to the transitional information contained within speech (e.g., Saffran et al., 1996, 1997), and from an early age, infants can use this co-occurrence information to calculate the likely locations of word boundaries in speech (Aslin et al., 1998; Teinonen et al., 2009; see Black & Bergmann, 2017, for a meta-analytic review). This process, termed *statistical learning*, has been investigated with speakers of a variety of languages (e.g. *German*: Marimon Tarter, 2019; Matzinger et al., 2019; *English*: Saffran et al., 1996; *Finnish*: Teinonen et al., 2009; *French*: Franco et al., 2015; and *Hebrew*: Siegelman et al., 2018). Moreover, TPs have been found to be informative in both directions, for both forwards (i.e., a subsequent syllable being predictable based on the preceding syllable, e.g., predicting “*by*” from “*ba*” in the word “*baby*”) and backwards transitions (e.g., predicting “*ba*” from “*by*”; Perruchet & Desaulty, 2008). Critically though, as a cue, TPs have significant language-specific properties. Notably, languages differ on whether forwards or backwards TPs are most informative (Onnis & Thiessen, 2013). In the current paper we determine the strength of both forwards and backwards TPs as cues to word identity in German.

### 1.3 Lexical and sublexical frequency

Frequency has been found to play an important role in language acquisition (see Ambridge et al., 2015, for a review). In natural language, word frequency follows Zipf’s law (Zipf, 1935, 1949), whereby a small number of words occur very frequently, whereas the vast majority of words are only rarely used. Zipfian distributions have been found to aid word segmentation in adult statistical learning studies, especially for larger lexica (Kurumada et al., 2013), presumably because highly frequent sequences enable rapid segmentation, which can act as anchors in subsequent utterances. This *anchor effect* has been found to benefit word segmentation in infant (Altvater-Mackensen & Mani, 2013; Bortfeld et al., 2005; Mersad & Nazzi, 2012; Shi & Lepage, 2008) and adult learners (Cunillera et al., 2010; Valian & Coulson, 1988), and in recent work, Cunillera et al. (2016) documented the neural signature of this effect—demonstrating that anchor words elicited greater stimulus-preceding negativity (a marker of expectation) in adults’ electroencephalography (EEG) data compared to less frequent words. Further support for the role of high frequency words in segmentation comes from the computational modeling literature; Monaghan and Christiansen (2010) demonstrated that their PUDDLE model of speech segmentation could quickly extract high frequency words from utterances contained within corpora of CDS, and use them to segment the remainder of the input.

Since frequency has been found to play a pivotal role in language acquisition, it follows that the benefits of highly frequent items may extend beyond word frequency, to the frequency of the syllables that words contain, and their syllabic structure. Syllable structures describe the patterns of consonants and vowels within a syllable (e.g., the syllable “ba” consists of a consonant and a vowel, abbreviated as a CV structure). These structures might follow a certain distribution, which might help segmentation in a similar way to the phonotactics of a language (see e.g., Boll-Avetisyan, 2018). That is, certain combinations of consonants and vowels might occur more often in specific positions and provide cues to word-hood.

### 1.4 Word length

For many of the world’s languages, the length of individual words can vary quite substantially. However, Zipf’s law (Zipf, 1935, 1949) states that word length is optimized for efficient communication, such that the most frequent words in a language are typically short. Support for this notion can be found for a range of languages, including English (see e.g., Li & Shirai, 2000, frequency counts for CDS corpora comprising 2.6 million words), Spanish (e.g., Alonso et al., 2011), and Swedish (Sigurd et al., 2004). In German, prior analysis revealed that approximately 50% of (written) words were monosyllabic, whereas around 30% were disyllabic, and approximately just 20% were longer still (Kaeding, 1897; Sigurd et al., 2004). While heterogeneity among word lengths is commonplace within language, a number of studies have demonstrated that having a variety of word lengths in speech poses a significant challenge to speech segmentation (Johnson & Tyler, 2010; Lew-Williams & Saffran, 2012; Kurumada et al., 2013; but see Perruchet & Vinter, 1998, for computational counter-evidence)—though this difficulty may be eased when speech contains additional cues (Frost et al., in press; Johnson & Tyler, 2010; Lew-Williams & Saffran, 2012). Conceivably, caregivers may remove some of the complexity associated with varying word length by providing a more uniform signal in CDS (Garmann et al., 2019; but see Segal et al., 2009). We investigated this possibility here.

### 1.5 Single-word utterances

Finally, another potential cue for identifying word boundaries is the occurrence of words in isolation, in single-word utterances. Research has found that most caregivers use single-word utterances in conversations with their infants, repeating around a third of these within close temporal proximity (Aslin et al., 1996; Brent & Siskind, 2001). Previous studies have estimated that up to 26% of utterances in English CDS comprise single words (Monaghan & Christiansen, 2010; but see MacWhinney & Snow, 1985, for a more conservative estimation of 14%). These single-word utterances have been suggested to help segmentation by first facilitating learning of these items (Junge et al., 2012), then flagging the boundaries of neighboring items in subsequent multi-word utterances (Peters, 1983; Pinker, 1984)—similar to the way in which high frequency words have been proposed to assist segmentation.

In the present study, we examined how many single-word utterances occurred in German CDS and how many single-word utterances were repeatedly produced, supposedly boosting the facilitated segmentation effect.

### 1.6 Aims and hypotheses

Past research has revealed that infants are sensitive to a range of cues to speech segmentation, and that the prevalence of these cues within the speech that children hear is subject to marked cross-linguistic variation. However, much remains to be done to determine the relative weighting of these cues across the world’s languages. In the current study, we adopt a corpus-based approach to determine cue availability in German CDS. Using High German as our target language, we took the equivalent of one day’s worth of input to a German-acquiring infant, and coded it for primary word stress, TPs, word frequency, word length, and the occurrence of words in single-word utterances. We hypothesized that we would find a dominant trochaic stress pattern for German similar to the one found in English (Cutler & Carter, 1987). In addition, we expected to see higher within-word than between-word TPs, and higher backwards than forwards TPs, similar to the results found in English (another right-branching language; Onnis & Thiessen, 2013). With regard to word frequency, we expected to find a Zipfian-like distribution of word types, word tokens, and syllables (Zipf, 1935, 1949), as has been found for a variety of the world’s languages, with a small number of words occurring with comparatively higher frequency than the remainder of words in the corpus. In terms of word length, we expected to find a greater proportion of shorter than longer words (Piantadosi et al., 2011; Zipf, 1935, 1949). Based on corpus analyses of English, we hypothesized that the corpus may contain a large proportion of single-word utterances (MacWhinney & Snow, 1985; Monaghan & Christiansen, 2010), with a large amount of these occurring repeatedly (Brent & Siskind, 2001; Monaghan & Christiansen, 2010).

## 2 Method

### 2.1 Data

Our data are openly available on the Open Science Framework (OSF): https://osf.io/vpdu6/. Our corpus comprised 20 German datasets from the Child Language Data Exchange System (CHILDES) database (MacWhinney, 2000). All datasets contained CDS spoken to children under two years of age. In order for our corpus to contain a representative sample of speech, we included files from a number of different children, recorded in different contexts (e.g., playing with toys, reading books, eating or bathing). This reduced the likelihood that speaker-specific patterns would influence our results. In total, we included data from 19 individual speakers talking to 10 different children, taken from the Caroline (Von Stutterheim, 2010), Manuela (Wagner, 2006), Miller (Miller, 1979), Rigol (Rigol, 2007), and Wagner (Wagner, 1974, 1985) corpora, with the age of the children at the time of recording ranging from 00;06.13 to 01;08.13 years. Together this totalled 07:32 hours of recording, during which caregivers (and occasionally siblings or researchers) provided 3967 utterances of CDS input, comprising an overall total of 16,474 words, and 14,660 words after filtering out proper names, sounds, and unintelligible speech (see the Appendix for further information on the included datasets). We estimate that this represents approximately one day’s worth of input.^1^

### 2.2 Coding

We coded the data by word tokens, that is, individual occurrences of words in CDS (so, with one entry for each of the 16,474 words). We defined a word as a unit that the child needs to segment to assign meaning.^2^ For each word, we coded for information at the word and syllable levels (for the full coding scheme see our OSF page). At the word level, we coded for word type (i.e., grouping different pronunciations of words, which result in different word tokens, into one word type), parts of speech (i.e., whether a word is a noun or a verb, etc.) and resulting categorization as content or function words, with open class words comprising nouns, verbs and adjectives, and closed class words comprising all remaining word categories. We also coded for word length (number of syllables), and word stress (the position of stress within words). At the syllable level, we coded the phonetic representation and syllable structure for each syllable of the word (i.e., describing the pattern of consonants and vowels which the syllable comprised, e.g., CV for the syllable [ba]). Sounds and unintelligible material were excluded from the analyses. Proper names were excluded from all analyses, except for the analysis which sought to establish the occurrence of proper names in single-word utterances.

## 3 Results

We will first outline the results for our analyses of word stress, TPs, and word and syllable frequencies. We will then present our findings for word length, and finally for those cues that can facilitate segmentation by flagging word boundaries (i.e., highly frequent words, single-word utterances). Our analyses and results are openly available on OSF: https://osf.io/vpdu6/. Additional analyses (including analyses on subsets of data, for instance, excluding monosyllabic words) can be found in the “Additional Analyses” section in our analysis file. All analyses were performed in R 3.6.3 (R Core Team, 2020).

### 3.1 Word stress

We examined the position of primary within-word stress, to establish how reliable the widely assumed dominant trochaic stress pattern is as a potential cue for segmentation in German. This analysis was performed on the whole corpus, excluding proper names and sounds.

The vast majority of words in our corpus of CDS were found to carry word-initial stress; in total, approximately 97% of words were stressed on the first syllable, whereas around 3% were stressed on the second, and less than 1% on the third to seventh syllables, but with no words stressed on the sixth syllable (see Table 1). In addition to this primary analysis, which used the entire corpus (thereby providing the closest approximation to the full input), we ran two further iterations—the first of which excluded repetitions (examining unique word tokens only), and the second of which was run on word tokens but excluded monosyllabic words (which can only be stressed on their first and only syllable). This was vital for establishing whether the observed stress pattern is generalizable, and is not reliant on particular tokens.

**Table 1.** Frequency of primary word stress at each syllable position.

Syllable position	Primary word stress: all word tokens		Primary word stress: unique word tokens		Primary word stress: word tokens excluding monosyllabic words	
	Count	%	Count	%	Count	%
1	14,206	96.90	1536	87.03	2771	85.92
2	398	2.71	191	10.82	398	12.34
3	47	0.32	31	1.76	47	1.46
4	6	0.04	5	0.28	6	0.19
5	1	0.01	1	0.06	1	0.03
6	0	–	0	–	0	–
7	2	0.01	1	0.06	2	0.06
8	0	–	0	–	0	–
Total	14,660		1765		3225	

For both of these iterations, we analyzed the resulting corpus in the same way as before. Both analyses yielded the same pattern of results: excluding repetitions, 87% of words were stressed on the first syllable, 11% on the second, and 2% on the third to seventh syllables (with no words carrying stress on the sixth syllable). Excluding monosyllabic words, 86% of words were stressed on the first syllable, 12% on the second, and 2% on the third to seventh syllables (again with no words carrying sixth-syllable stress). Thus, these data provide strong evidence to suggest that German CDS has a dominant trochaic stress pattern (i.e., word-initial stress).

### 3.2 Transitional Probabilities

We next examined the way in which TPs between syllables varied according to two key aspects: *context* (i.e., for transitions *within* versus *between* words); and *direction* (i.e., probabilities of syllable co-occurrence for *forwards* versus *backwards* transitions). To do this, we extracted pairs of syllables from either within or between words within utterances only (i.e., not crossing utterance boundaries, which are typically indicated by a pause or a switch in speakers), and calculated forwards and backwards TPs for both contexts. Forwards TPs were calculated following Equation (1a), and backwards TPs following Equation (1b). That is, the forwards TPs within the word “baby,” for instance, were calculated by dividing the number of times the two syllables “ba” and “by” co-occurred by the total number of times the syllable “ba” occurred:

(1a) $$\mathrm{probability}\;\mathrm{of}\;B,\;\mathrm{given}\;A=\frac{\#\;\mathrm{occurrences}\;A+B}{\#\;\mathrm{occurrences}\;A}$$

(1b) $$\mathrm{probability}\;\mathrm{of}\;A,\;\mathrm{given}\;B=\frac{\#\;\mathrm{occurrences}\;A+B}{\#\;\mathrm{occurrences}\;B}$$

**Figure 1.**
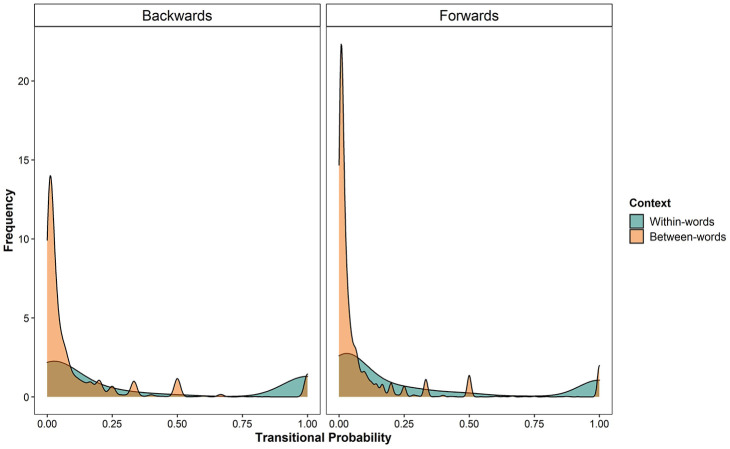
Density plot of transitional probabilities (TPs) between syllables in the corpus. The panels on the left and right show the frequency data for backwards and forwards transitions, respectively. TPs within words are indicated in green, whereas TPs between words are indicated in orange.

Figure 1 shows that both backwards and forwards TPs are higher within than between words. To test whether the TPs varied according to context and direction, we fitted a linear mixed-effects model using the *lme4* 1.1-23 package (Bates et al., 2015). The dependent variable was TP, and context and direction were entered as fixed effects. We used deviation contrasts for context (*within-word*: -0.5, *between-word*: 0.5) and direction (*forwards*: -0.5, *backwards*: 0.5). We fitted the maximal model supported by the data (Barr et al., 2013), controlling for the syllable pair as a random intercept with direction^3^ as a random slope. To examine the effects of the model predictors, we used likelihood-ratio (*χ*^2^) comparisons to obtain *p*-values (through serial decomposition), and bootstrap simulations (Runs = 1000) to calculate 95% confidence intervals for the beta estimates. The marginal and conditional *R*^2^ effect sizes are also reported as goodness-of-fit estimates. These denote the proportion of the variance explained by the model both with (conditional *R*^2^) and without (marginal *R*^2^) controls for sources of random variance (Johnson, 2014; Nakagawa & Schielzeth, 2013; Nakagawa et al., 2017).

There was a significant main effect of context, with TPs being higher within words than between (*within words*: *M* = 0.33, *SD* = 0.41; *between words: M* = 0.11, *SD* = 0.21). There was also a significant effect of direction, with TPs being higher for backwards than forwards transitions (*backwards: M* = 0.17, *SD* = 0.29; *forwards: M* = 0.13, *SD* = 0.26; see Figure 1 and Table 2). There was a significant interaction between context and direction, driven by a larger difference between the two contexts for the backwards TPs (*forwards: within words: M* = 0.30, *SD* = 0.39; *between words: M* = 0.10, *SD* = 0.21; *backwards: within words: M* = 0.36, *SD* = 0.44; *between words: M* = 0.12, *SD* = 0.22). The maximal model with context and direction as fixed predictors accounted for approximately 10% of the variance in the data without the random effects structure, and 46% of the variance with the random effects structure.

**Table 2.** Summary of the linear mixed-effects model for transitional probabilities (TPs).

	Estimate	95% confidence interval	Standard error	χ^2^	*df*	*p*
(Intercept)	0.220	[0.215, 0.225]	0.003	–	–	–
Context	−0.219	[−0.229, −0.209]	0.005	1556.53	1	< 0.001
Direction	0.047	[0.035, 0.059]	0.006	52.46	1	< 0.001
Context × direction	−0.041	[−0.063, −0.018]	0.012	11.24	1	< 0.001

*Notes*: context distinguished between within-word and between-word TPs, direction distinguished between forwards and backwards TPs, deviation-coded as: within-word, -0.5; between-word 0.5; forwards, -0.5; and backwards, 0.5; model fit: Bayesian information criterion (BIC) = 2211; modified Akaike information criterion (AICc) = 2151; $R_{m}^{2}$ = 0.098; and $R_{c}^{2}$ = 0.464.

### 3.3 Frequency

#### 3.3.1 Word frequency

We examined the frequency distribution of words in the input, in the light of the suggestion that highly frequent words and a Zipfian-like distribution (Zipf, 1935, 1949) can support segmentation (Kurumada et al., 2013).

**Figure 2.**
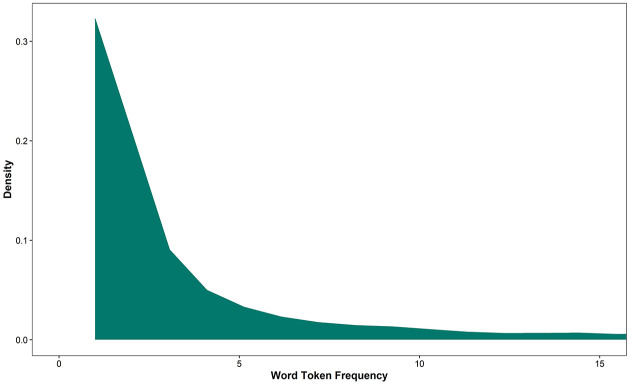
Density plot of word token frequencies, indicating the extent to which words occur with particular frequencies in the corpus.

There was a Zipfian-like frequency distribution (Zipf, 1935, 1949) for both word tokens and word types (see Figure 2 for a density plot of word token frequencies; see our OSF repository for the same plot but with word types), with the corpus containing a large amount of low frequency words (i.e., open class words such as nouns, which were high in quantity, but were rarely repeated, amounting to 31% of words in the corpus), and a small amount of words with much higher frequencies (i.e., closed class words such as determiners, which were used in combination with all nouns, e.g., *ein Fuchs* “a fox,” *ein Häschen* “a rabbit,” and *ein Laster* “a truck,” amounting to 69% of words in the corpus). This was in line with our hypothesis. A summary of the word frequency density (i.e., the percentage of individual words in the corpus occurring once, twice, three times, etc.) is provided in Table 3, alongside the analogous results from Kaeding’s (1897) study of written German. Both studies revealed the same Zipfian-like frequency distribution (i.e., in both sets of input, approximately half of the words occurred just once; 49% of words in Kaeding’s study, 50% of words in the current corpus; and approximately 15% of words occurred twice, etc.).

**Table 3.** Summary of word token frequencies in the present corpus of German child-directed speech, and in Kaeding’s (1897) study of written German.

Word token frequency	Kaeding (1897)	Current dataset
1	49.14%	49.76%
2	13.37%	15.12%
3	6.61%	7.65%
4	4.31%	4.34%
5	3.04%	3.37%
6–10	7.76%	7.47%

**Figure 3.**
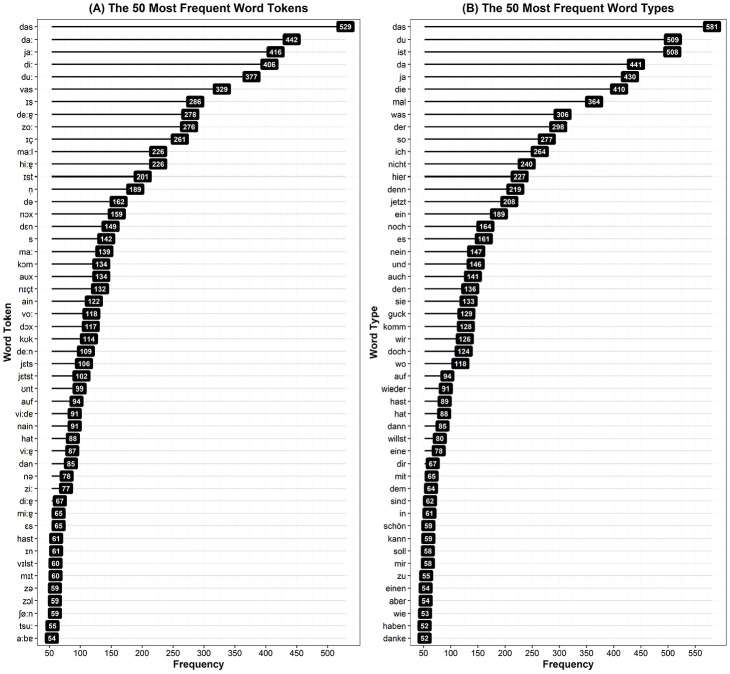
Frequencies for the 50 most frequent words in the corpus. Panel A (left) shows word tokens, and Panel B (right) shows word types.

We focused our subsequent frequency analyses on the 50 most frequent items (computing analyses for both word tokens and word types), to shed light on the properties of the words that infants were hearing the most. For word tokens, the 50 most frequent items constituted 54% of the corpus (7896 out of 14,660 words; see Figure 3 Panel A), and were almost exclusively monosyllabic, with *wieder* (“again,” 91 occurrences) and *aber* (“but,” 54 occurrences) being the only multi-syllabic exceptions. For word types, the 50 most frequent items constituted 59% of the corpus (8602 out of 14,660 words; see Figure 3 Panel B). As with word tokens, the vast majority of word types were monosyllabic, with six exceptions; *wieder* (“again,” 91 occurrences), *eine* (“a,” 78 occurrences), *aber* (“but,” 54 occurrences), *einen* (“a,” 54 occurrences), *danke* (“thanks,” 52 occurrences) and *haben* (“have,” 51 occurrences), which were all disyllabic. Thus, these data suggest that the vast majority of the most frequent words in German CDS are monosyllabic, with a small number of disyllabic exceptions.

To investigate which *kind* of words were most frequent, we distinguished between function and content words. Of the 50 most frequent words, 41 tokens (82%) or 39 types (78%) were function words (e.g., *das* “the” or *ja* “yes”), whereas just 9 tokens (18%) or 11 types (22%) were content words (e.g., *guck* “look” or *schön* “nice”). These highly frequent function words accounted for approximately 47% of word tokens in the entire corpus (6834/14,660), and 50% of word types (7290/14,660). The vast majority were monosyllabic (39/41 tokens, and 35/39 types). These highly frequent monosyllabic function words accounted for approximately 46% of word tokens in the entire corpus (6689/14,660), and 48% of word types (7039/14,660).

#### 3.3.2 Syllable and syllable structure frequency

**Figure 4.**
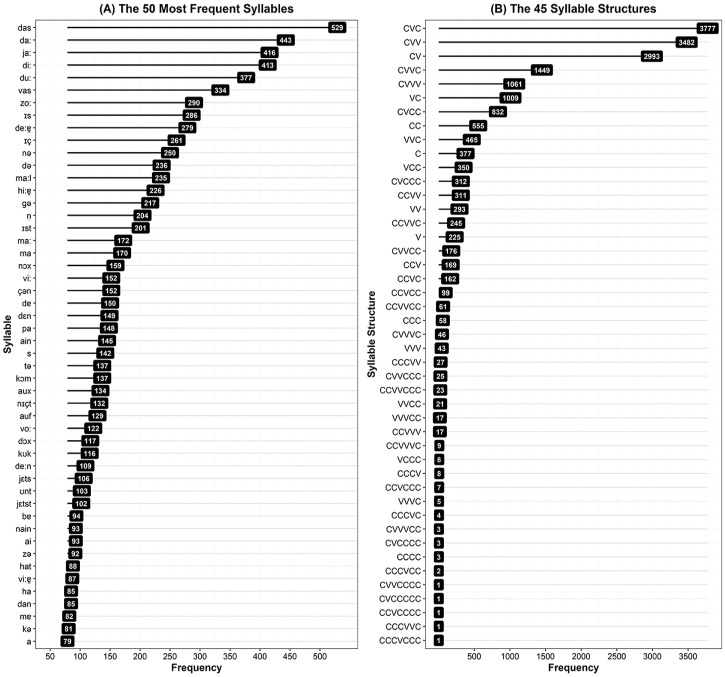
Syllable and syllable structure frequencies in the corpus. Panel A (left) shows the 50 most frequent syllables, and Panel B (right) shows all 45 different syllable structures. Because we consider syllabic consonants such as [ṇ] as consonants, it is possible to have syllables with multiple consonants but no vowels (e.g., the second syllable of the verb *putzen* [pʊtsṇ] “clean” consists of three consonants); similarly, because we code long vowels as VV, it is possible to have syllables with multiple vowels (e.g., the word *er* [e:ɐ̯] “he” consists of three vowels).

We examined the frequencies of individual syllables, and particular syllable structures. For instance, the word *Baby* consists of two syllables, [be:] and [bi] with the respective syllable structures CVV and CV. Our corpus comprised 18,736 syllable tokens in total, and particular syllables were seen to occur with a Zipfian-like distribution (Zipf, 1935, 1949; see OSF for a density plot of syllable frequencies). Because of the large quantity of monosyllabic words within the corpus, the most frequent syllables were identical to the most frequent word tokens (see Figure 4 Panel A). Of particular interest, then, are syllables occurring in multisyllabic words. The results of our additional analyses excluding monosyllabic words, as well as focusing particularly on disyllabic and trisyllabic words (as used in most artificial language learning studies) can be found on OSF in Section 5 of the analysis file. Since children, however, encounter the monosyllabic words in their input, we draw our conclusions from the complete dataset, reporting only the results for the whole corpus (including all word lengths) here. We summarize our findings for multisyllabic words, as well as disyllabic and trisyllabic words in the Supplementary Material folder on OSF.

For syllable structure, there was again a Zipfian-like distribution (Zipf, 1935, 1949; see OSF for a density plot of syllable structure frequencies), with a small number of structures occurring much more frequently than others. We examined the frequencies with which particular syllable structures occurred at different positions within words, to explore the possibility that patterns of regularity may indicate word boundaries (for instance, if certain structures are mostly found at word edges).

There were 45 different syllable structures within our corpus (see Figure 4 Panel B). In initial and final positions, there were 42 different structures; in medial positions, there were 24. We observed slight differences dependent on syllable position; the most common structure in medial positions was an open syllable (CV as in [gə]; comprising 40% of medial syllables), whereas the most common structures in initial and final positions were closed (CVV as in [da:] or CVC as in [das]). Word-initial syllables ended most often in a long vowel (i.e., CVV; comprising 22% of initial syllables), whereas syllables in word-final positions ended most often in a consonant (i.e., CVC; comprising 23% of final syllables). However, these three structural types (CV, CVV, and CVC) were found to occur in all positions within words with a high degree of frequency, constituting the most frequent syllable structures for all three locations—limiting the extent to which these structures may serve to cue segmentation. The difference between structure occurrence in initial versus final positions is particularly subtle (*initial*: CVV 22%, CVC 21%; *final*: CVV 18%, CVC 23%), possibly because of the large amount of monosyllabic words in the corpus. Again, syllable structures occurring in multisyllabic words can provide further insights. We summarize our findings for multisyllabic words, as well as disyllabic and trisyllabic words in the Supplementary Material folder on OSF.

### 3.4 Word length

Next, we examined word length, and the frequency with which different word lengths occurred in the input. Table 4 lists this for the number of word tokens, the number of unique word tokens, and the number of unique word types. Word tokens provided the raw frequency counts of every word in the corpus. Unique word tokens represent the number of different words in the corpus regardless of the number of repetitions of this item (e.g., a list containing: “one, one, two” would count three word tokens but only two unique word tokens). The unique word types column combines different pronunciations of the same word (e.g., a list containing: “not, not, n’t,” would count three word tokens, two unique word tokens but only one unique word type). We computed the word length of unique word tokens and unique word types as a measure of robustness to ensure the reliability of the findings and to control for potential correlations with other effects such as word frequency.

**Table 4.** Frequency statistics for word length (measured in number of syllables).

Number of syllables	Number of word tokens		Number of unique word tokens		Number of unique word types	
	Count	%	Count	%	Count	%
1	11,435	78.00	655	37.09	553	34.33
2	2550	17.39	715	40.49	672	41.71
3	545	3.72	293	16.59	285	17.69
4	98	0.67	78	4.42	76	4.72
5	21	0.14	17	0.96	17	1.06
6	9	0.06	7	0.40	7	0.43
7	0	–	0	–	0	–
8	2	0.01	1	0.06	1	0.06
Total	14,660		1766		1611	

The words in our corpus were between one and eight syllables long (with the longest words being nominal compounds). In total, 11,435 (78%) of all words were monosyllabic, 2550 (17%) disyllabic, 545 (4%) trisyllabic, and 130 (1%) between four and eight syllables long (with seven-syllable words never occurring). After controlling for frequency (via excluding repetitions) there was a slight shift in this pattern, with disyllabic words occurring slightly more often (40%) than monosyllabic words (37%), followed by trisyllabic words (17%), and words with four to eight syllables (6%). This pattern shift indicates that shorter (monosyllabic) words were subject to a greater degree of repetition in the corpus. Interestingly, although German allows significant compounding, only 2% of word tokens in our corpus were compounds.

### 3.5 Single-word utterances

Finally, we examined the corpus for single-word utterances, which may aid segmentation by subsequently flagging the boundaries of adjacent words in multi-word utterances. Of the 3513 utterances (excluding proper names and sounds), 527 utterances (or 15%) comprised single words (898 of 3967 utterances, or 23%, including proper names and sounds). Although we excluded proper names and sounds from all of our prior analyses, proper names–particularly the child’s—have been found to be highly salient anchors for infants’ segmentation of multi-word utterances (Bortfeld et al., 2005). Thus, we examined how often proper names occurred in single-word utterances. Across the whole corpus, single-word utterances comprising proper names occurred just 42 times—amounting to 7% of single-word utterances, and 1% of all utterances (including proper names, but excluding sounds).

The remainder of the single-word utterances were found to largely comprise function words (71% of single-word-utterances excluding proper names and sounds). The most frequent words were particles such as *ja* (“yes”), which amounted to 17% of single-word utterances (3% of all utterances in the corpus), *nein* (“no,” 6% of single-word utterances), *danke* (“thanks,” 4% of single-word utterances), and *bitte* (“please,” 3% of single-word utterances), adverbs such as *so* (“like this,” 10% of single-word utterances), and *da* (“there,” 9% of single-word utterances), the pronoun *was* (“what,” 5% of single-word utterances), and the interjection *hallo* (“hello,” 2% of single-word utterances). 21% of single-word utterances were content words such as the imperative *komm* (“come,” 3% of single-word utterances), and the noun *Baby* (“baby,” 3% of single-word utterances). A list of all single-word utterances, including the remaining ones which occurred less than 10 times, can be found in the Supplementary Material folder on OSF.

## 4 Discussion

This study offers the first corpus analysis investigating the availability of word segmentation cues in German CDS, and the first to combine an analysis of a broad range of possible cues. We analyzed approximately one day’s worth of input data from the CHILDES database (MacWhinney, 2000), examining a variety of potential word segmentation cues in German CDS: word stress, TPs, word and syllable frequencies, syllable structures, word length, and single-word utterances. We discuss the results for each of the cues in turn.

### 4.1 Word stress

Analyses of the corpus revealed a dominant and reliable trochaic stress pattern, with almost all words (97%) being stressed on the first syllable—providing strong evidence for the widely assumed trochaic stress pattern in German (Friederici et al., 2007; Höhle et al., 2001, 2009; Tippmann et al., 2015; Weber et al., 2004). Crucially, the trochaic stress pattern persisted even when monosyllabic words were withheld from the analysis—indicating that infants may be able to use stress to inform segmentation of words of various lengths. These findings indicate that word stress may be an even stronger cue in German than English, where 90% of words were found to contain word-initial stress (Cutler & Carter, 1987).

In the German linguistics literature there is still somewhat of a controversy about the rules underlying the predominant stress pattern in German, with some researchers claiming a universal rule assigning stress from the right word-edge (e.g., Giegerich, 1985; Vennemann, 1990; Wiese, 1996), and others claiming a different rule for stress assignment in words of Germanic origin (word-initial stress) versus more recent borrowings (right-edge stress) (Benware, 1980; Braches, 1987; Féry, 1986; Wurzel, 1970, 1980; see Goedemans & van der Hulst, 2013a, 2013b for a classification; and Jessen, 1999 for a discussion). We note, though, that since 95% of the words in our corpus were monosyllabic or disyllabic, establishing whether primary stress occurred on the first versus the penultimate syllable would not be possible. That is, for infants segmenting the speech detailed in our corpus, both of these possible stress patterns would be interpreted as containing word-initial stress.

Nevertheless, given the data reported here, we can assume that German CDS largely adheres to a word-initial stress pattern, which children can draw upon with high return given its ubiquity in the input (potentially with a small number of exceptions due to affixation—3% in our corpus). This is consistent with experimental work on infant segmentation, which has shown that children make use of stress cues early in development (e.g., Höhle et al., 2001; Houston et al., 2000).

### 4.2 Transitional Probabilities

Analysis of TPs provided support for another cue to word segmentation in German CDS, with TPs being significantly higher within than between words. This finding builds on prior demonstrations that TPs are informative cues to word-hood in a variety of languages (e.g., Saffran, 2003; Saksida et al., 2017)—extending this to German. Together with the many experimental demonstrations of TP-based segmentation in experiments (Saffran et al., 1996; see Black & Bergmann, 2017, for a review), the naturalistic data lend credence to the possibility that infants draw on these statistics during language acquisition.

There are two additional features of the TP results that deserve discussion. The first concerns the magnitude of within-word TPs, which appear to be rather small compared to experimental studies, where words are typically defined by TPs that are much higher (indeed, in psycholinguistic experiments these are often perfect, i.e., TP = 1.0). Thus, if children draw on TPs to aid segmentation “in the wild,” they are doing so in a much noisier channel. Nevertheless, there is good reason to believe that they do. For instance, Pelucchi et al. (2009) showed that 8-months-old infants can segment words from a foreign natural language (English-acquiring infants segmenting Italian speech) under experimental conditions following a short exposure phase.

The quasiregular nature of this cue is a necessary outcome of the generative nature of language, and might actually be a key to learning. Kidd et al. (2012) argue that infants demonstrate a “Goldilocks” effect, such that they prefer to attend to events that are neither highly predictable nor unpredictable, thus avoiding making generalizations that are either too simple or too complex. A recent computational model of word learning suggests that cue variability may indeed serve to help, rather than hinder, learning—guiding the creation of a robust, canalized language system that is resistant to noise in the input (Monaghan, 2017). This possible utility of noise in learning is underpinned by the principle that variation in the availability and reliability of distributional cues may encourage learners to seek guidance from multiple possible information sources, reducing the importance of a particular individual cue, and increasing the resilience of the language system to noise. Variability within various distributional statistics has been found to have advantages for segmentation (Kurumada et al., 2013), word learning (Hendrickson & Perfors, 2019; Monaghan et al., 2017), semantic category learning (Lany, 2014), and acquisition of syntactic structure (Gómez, 2002). Here, we raise the possibility that this may also extend to variability among TPs.

Interestingly, we found backwards TPs to be significantly higher (and thus, more informative) than forwards TPs, and the magnitude of the difference between within-word and between-word TPs was larger in the backwards direction. This finding provides further support for the notion that TPs are informative in both directions, as has been observed in English (Perruchet & Desaulty, 2008). Further, these data lend critical support to the idea that backwards TPs are more informative than forwards TPs in right-branching languages such as English (Onnis & Thiessen, 2013)—extending this to German. This is in contrast to the distributional patterns observed for left-branching languages such as Korean, where forwards TPs are held to be more informative (Onnis & Thiessen, 2013). The generalization here is that there are distinct influences of typology on probabilistic distributions in language; in particular, head-direction creates conditions in which one prominent element grounds a dependent one (e.g., compare *red wine* in English to *vino rosso* in Italian). Ultimately, this means that any statistical learning mechanism useful for segmentation must rapidly attune to the target language (Onnis & Thiessen, 2013).

Taken together, the results concerning stress and TPs suggest that, in German, stress is a dominant segmentation cue; consistent with recent experimental work by Marimon Tarter (2019), who found that German-acquiring 6-month-old infants and German-speaking adults preferentially attended to stress over statistical information during a segmentation task. This is the opposite pattern than has been observed for English (Thiessen & Saffran, 2003), suggesting an unexpected crosslinguistic difference in the two, highly-related, languages. Further research will be necessary to unpack these differences.

### 4.3 Lexical and sublexical frequency

With regard to frequency, we found Zipfian-like distributions (Zipf, 1935, 1949) for every feature that we analyzed; words, syllables, and syllable structures, replicating Kaeding’s (1897) work on written German. These findings provide further evidence for the well-established ubiquity of Zipfian distributions in natural language. In recent work, such distributions have been suggested to help speech segmentation (Kurumada et al., 2013). In terms of word frequency, highly frequent items have been proposed to aid segmentation by acting as anchor points for subsequent segmentation to occur around; these words are believed to undergo early extraction from the speech stream, before flagging the boundaries of the words they appear alongside in subsequent speech (Altvater-Mackensen & Mani, 2013; Bortfeld et al., 2005; Kurumada et al., 2013; Mersad & Nazzi, 2012; Monaghan & Christiansen, 2010; Shi & Lepage, 2008). The precise utility of Zipfian distributions among syllables and syllable structures remains to be established; however, it is conceivable that these may serve segmentation in a similar way. This possibility requires empirical investigation.

### 4.4 Word length

Regarding word length, we found the majority of words to be monosyllabic, with only 22% of words having more than one syllable, and only 5% of words having more than two syllables. The amount of monosyllabic words reported here was considerably higher than that described in previous reports on German (78% here, versus 50% in Kaeding, 1897—which Zipf’s calculations were based upon). This discrepancy may be traced back to the contrast between spoken and written language, with spoken language shortening words by the use of contractions; or the difference may be due to the contrast between child-directed and adult-directed speech, with CDS potentially being defined not only by the use of a higher pitch and shorter utterances (e.g., Cristia, 2013), but also by the use of shorter words in general (Garmann et al., 2019; but see Segal et al., 2009). This resulted in a much larger proportion of monosyllabic words here than in Kaeding’s (1897) frequency dictionary (though a comparison of our data with data for more recent adult-directed speech, collected in a similar manner, would be necessary to draw more firm conclusions). In any case, data from our corpus indicate that caregivers may optimize word length (via simplification) for efficient communication to a greater extent in child—compared to adult—directed speech (see Garmann et al., 2019), with even more monosyllabic words than would be predicted by Zipf’s law (Zipf, 1935, 1949). This in turn offers an interesting new perspective on the finding that a variety of word lengths adds difficulty to segmentation (Johnson & Tyler, 2010; Lew-Williams & Saffran, 2012). It appears that, if word length is a problem for segmentation, for German infants, this may well be circumvented by a fairly uniform input consisting of mostly monosyllabic words (see Perruchet & Vinter, 1998, for computational evidence in support of this proposal).

Our analyses of word length also provide another instance where cues appear to converge. We found the vast majority of the 50 most frequent words, and almost two-thirds of the whole corpus to be monosyllabic function words. Importantly, those words are more stressed in German than in English, and therefore perfectly detectable by the infant (Höhle & Weissenborn, 2003; but see also Gerken, 1994; Gerken & MacInthosh, 1993; Shafer et al., 1992, for evidence of the detection of function words in English). In consequence, infants can detect and segment those highly frequent function words, and subsequently, use them as anchors to facilitate acquisition of the words surrounding them (Bortfeld et al., 2005; Mersad & Nazzi, 2012).

### 4.5 Single-word utterances

Finally, 15% of the utterances in our corpus were single words, 85% of which were words that were repeated in isolation at least once, and 62% occurred in isolation between 10 and 90 times. The amount of single-word utterances in German CDS is similar to that observed for English CDS, although it falls toward the lower boundary of the estimations made in prior research (estimated at around 14% by MacWhinney & Snow, 1985; and 26% by Monaghan & Christiansen, 2010). Nevertheless, this yields a fairly substantial amount of isolated words, which can potentially be segmented more easily, and in turn subsequently aid segmentation of adjacent words in multi-word utterances (Peters, 1983). Previous research found that approximately 33% of single-word utterances were repeated in close temporal proximity (Brent & Siskind, 2001). Though we did not examine temporal proximity here, we can add that 85% of single-word utterances were indeed repeated within the corpus.

In addition, we found that 1% of all utterances comprised proper names presented in isolation. This was mostly the child’s own name (74%), but also included names of siblings (17%), and others (9%). The number of occurrences concerning children’s names here is comparable to prior observations in English CDS (1%, Monaghan & Christiansen, 2010), and accounts for 20% of all the times a child’s name occurred in the speech (compared to 24% of instances in English; Monaghan & Christiansen, 2010). These isolated occurrences of names may help increase their prominence to young learners, with names being suggested to enjoy a privileged position as salient anchor words that lend significant benefits to segmentation (Bortfeld et al., 2005; Mersad & Nazzi, 2012), operating in a similar way to high frequency words. Thus, these findings indicate that single-word utterances (Brent & Siskind, 2001; Monaghan & Christiansen, 2010), and particularly isolated incidences of children’s names, may serve segmentation to a similar degree in German CDS as has been previously suggested for other languages.

### 4.6 Limits and future directions

Despite addressing a broad variety of segmentation cues, there are a number of potential cues not addressed here that may be valuable during language acquisition. For instance, we did not assess phonotactics or allophonic variation. Future assessments may wish to include these features to provide an extensive overview of the potential segmentation cues in German CDS. Additionally, while our results paint a strong picture of the prevalence of several individual cues in German CDS, indicating their potential importance for speech segmentation, determining how these cues interact requires further exploration. Moreover, establishing the way in which learners draw on these cues together during learning requires much empirical investigation. One way to address this topic is to combine cross-linguistic research, including corpus studies as well as experimental studies, with computational modeling approaches (cf. Monaghan & Rowland, 2017).

We note too that the syllable serves as the segmentation unit for many of these cues, which raises the question of how infants come to identify the precise boundaries of a given syllable (which would be necessary in order for it to inform subsequent learning). This capability is likely the outcome of several distinct, and perhaps converging, sources of information—such as the phonotactics of a language, in addition to its prosody, as well as broader distributional properties (e.g., permissible syllable structures and TPs). Since the majority of words in our corpus of German CDS were monosyllabic and stressed word-initially, it is difficult to speculate on the relative contributions of other cues for this task, but this would be an insightful avenue for future research.

We also acknowledge that our results are based on what may be considered a relatively small amount of data, particularly given the recent surge in studies using day-long recordings (e.g., Casillas et al., 2020; Donnelly & Kidd, in press; Weisleder & Fernald, 2013). However, there is evidence to suggest that corpus size does not lead to significant changes in distributional statistics (see Gambell & Yang, 2006; and see Saksida et al., 2017, for TP analyses on nine different languages using similar sized corpora). Thus, it is unlikely that the results we observed would vary significantly with a larger corpus. We note, too, that the kinds of in-depth, fine-grained analyses we conducted are atypical of studies using day-long recordings, which are based on fairly course estimates of language, computed via automated algorithms or through transcription of small subsets of the data. Rather, our focus on the minutiae of lexical and sublexical distributional information required a good degree of hand-coding.

Finally, it is important to acknowledge that we are generalizing over a large age range. While we restricted our analyses to speech directed at infants aged 6 to 20 months, it is likely that at least some properties of CDS change across this time frame (see e.g., Kunert et al., 2011; Raneri et al., 2020; Vosoughi & Roy, 2012). For instance, Kunert et al. (2011) reported evidence to suggest that two of the cues investigated in the current study (syllable structure and word length) become more complex in English CDS as children get older and start to use more complex syllables and words themselves. Therefore, longitudinal research of the type we have reported here would be a valuable addition to the literature.

## 5 Conclusion

We conducted the first corpus analysis investigating a broad range of word segmentation cues in German CDS, finding a highly reliable word-initial stress pattern, higher within-word and backwards TPs, and a Zipfian-like distribution (Zipf, 1935, 1949) of word and syllable frequencies. We also found slight differences of syllable structures between positions within a word, a prevalence of monosyllabic words, and especially highly frequent, short function words, and finally, a significant amount of single-word utterances. All of the cues we examined have the potential to aid word segmentation, and of course, might boost the effect when infants can draw on a combination of cues, as is the case in natural language (Brent & Cartwright, 1996; Matzinger et al., 2019).

## Appendix: Metadata on the Child Language Data Exchange System (CHILDES) corpora

**Table A1.** Background information on the data included in the corpus.

Corpus	Child	Age	Length of recording	Total number of utterances	Total number of utterances excluding unintelligible utterances	Speaker	Number of utterances per speaker	Number of utterances per speaker excluding unintelligible utterances
Caroline	Caroline (female (f))	00;10.01	00:06:44	22	22	MOT	22	22
		00;10.02	00:06:37	20	20	MOT	20	20
		00;11.25	00:18:22	81	79	MOT	63	63
						FAT	18	16
		01;00.19	00:11:04	37	37	MOT	37	37
		01;00.23	00:16:37	69	69	MOT	53	53
						FAT	16	16
		01;01.02	00:12:06	61	61	MOT	61	61
		01;01.04	00:01:46	10	10	MOT	10	10
Manuela	Dasca (male (m))	00;06.13	00:00:34	5	5	MOT	5	5
	Nibra (m)	00;10.24	00:00:24	5	4	MOT	5	4
	Oskoa (m)	00;10.12	00:01:05	28	28	MOT	28	28
		00;10.12	00:00:50	18	18	MOT	18	18
	Viala (m)	00;06.13	00:01:08	21	21	MOT	21	21
	Viwia (m)	00;10.20	00:00:15	7	7	MOT	7	7
Miller	Kerstin (f)	01;03.22	Approximately 00:15:00*	214	211	MOT	199	198
						FAT	13	11
						OBS	2	2
		01;04.13	Approximately 00:30:00*	421	416	MOT	284	283
						FAT	22	18
						OBS	79	79
						VIS	36	36
Rigol	Corinna (f)	01;00.09	00:31:22	714	657	MOT	379	356
						FAT	322	289
						OBS	13	12
		01;00.23	00:32:38	629	577	MOT	279	257
						FAT	348	318
						OBS	2	2
		01;01.08	00:33:44	487	479	MOT	463	455
						OBS	24	24
	Cosima (f)	01;08.13	00:30:38	449	443	MOT	354	350
						FAT	14	12
						OBS	81	81
Wagner	Katrin (f)	1;05.15	03:22:00	855	803	MOT	607	570
						FAT	248	233
Total			07:32:54	4153	3967			

*Notes*: the Caroline corpus contains utterances consisting of several sentences which makes the utterances longer than the ones in the other corpora, that is, to work with comparable numbers the number of utterances in the Caroline corpus should be increased; * the length of recording of the Miller corpus is an estimate based on the number of utterances in the datasets; the Speaker abbreviations are: MOT = mother, FAT = father, OBS = observer/researcher, VIS = visitor.

## Notes

1. A recent study by Donnelly and Kidd (in press) using daylong recordings found that, at 12 months, the average number of words a child hears is 14,572 (SD = 6826), based on a large sample of over 100 children acquiring Australian English as a first language. This number increases slightly across the next year, to 16,827 words at 24 months. Note that this estimates the words in the environment, only a subset of which is likely to be child-directed speech.

2. This is different from a phonological word as those comprise chunks such as *haste* for *has(t) de [: du]* (‘have you’), which we treated as two different words.

3. Direction is included as a random slope because forwards and backwards transitional probabilities (TPs) are calculated for each syllable pair. Context, however, is not included as a random slope because within-word and between-word TPs are almost exclusively calculated on different syllable pairs.
